# Prognostic value of sarcopenia in patients with liver cirrhosis: A systematic review and meta-analysis

**DOI:** 10.1371/journal.pone.0186990

**Published:** 2017-10-24

**Authors:** Gaeun Kim, Seong Hee Kang, Moon Young Kim, Soon Koo Baik

**Affiliations:** 1 Department of Nursing, Keimyung University, Daegu, Republic of Korea; 2 Department of Internal Medicine, Yonsei University Wonju College of Medicine, Wonju, Republic of Korea; 3 Cell Therapy and Tissue Engineering Center, Yonsei University Wonju College of Medicine, Wonju, Republic of Korea; 4 Institute of Evidence based Medicine, Yonsei University Wonju College of Medicine, Wonju, Republic of Korea; Taipei Veterans General Hospital, TAIWAN

## Abstract

**Background:**

Sarcopenia is a common syndrome in chronic diseases such as liver cirrhosis. The association between sarcopenia and outcomes, such as complications and survival has recently been described in various patient groups. However, study results remain inconclusive. Therefore, the aim of this study was to systematically review the impact of sarcopenia on outcome in patients with cirrhosis.

**Methods and findings:**

We conducted a systematic review (SR) and meta-analysis (MA) on the impact of sarcopenia on outcome in liver cirrhosis was performed according to the Preferred Reporting Items for Systematic Reviews and Meta-Analysis guidelines. Of the 312 studies identified, 20 were eligible according to our inclusion criteria. Most of the studies used CT to diagnose sarcopenia. Two studies used bioelectrical impedance analysis (BIA), 10 studies used skeletal muscle index (SMI) and 8 studies used total psoas muscle area (TPA). Seven studies included Asian participants and the remaining 13 studies included Western participants. The prevalence rate of sarcopenia among participants was mean 48.1%, and appeared more among men with a rate of 61.6% whereas the rate was 36% for women. With respect to clinical outcomes, patients with sarcopenia had poorer survival rates and an increased risk of complications such as infection compared to those without sarcopenia. According to the analysis of race subgroup, Asians had a HR 2.45 (95% confidence interval (CI) = 1.44–4.16, *P* = 0.001) of mortality whereas Westerners had a HR 1.45 (95% CI = 1.002–2.09, *P*<0.05).

**Conclusions:**

Based on this SR and MA, the presence of sarcopenia is related to a poor prognosis and occurrence of cirrhotic complications and could be used for risk assessment. Moreover, Asian participants had higher mortality related to sarcopenia compared to the Western participants.

## Introduction

Cirrhosis is a leading cause of mortality worldwide, and it is associated with a significant reduction in health-related quality of life. [1,2] The ultimate therapy for liver cirrhosis is liver transplantation (LT). [3–5] Predicting the evolution of liver cirrhosis to improve therapeutic decision is a challenge, especially for patients who can obtain a donor liver because it is resource-spending. Therefore, prognostic factors that can be used to determine survival without LT are required. Currently, the Child-Turcotte-Pugh (CTP) score and the Model for End Stage Liver Disease (MELD) scores are known as the best tools for predicting mortality in patients with cirrhosis. The CTP score is simply obtained from clinical and current laboratory data. [6] The MELD score was initially created to predict survival in patients with complications of portal hypertension undergoing elective placement of transjugular intrahepatic portosystemic shunt (TIPS). [7] Despite certain advantages of the MELD and CTP scores, the major limitation of these scores is the lack of evaluation of the nutrition and functional status of patients with cirrhosis. However, estimation of the nutritional status in patients with cirrhosis is difficult because of fluid collection caused by impaired protein synthesis. [8] Therefore, objective assessment of nutritional status needs to be established in cirrhotic patients.

Sarcopenia is a syndrome characterized by progressive and generalized loss of skeletal muscle mass and strength, shown to be prevalent in adults with cancer and common chronic comorbidities such as liver cirrhosis. [9] In such patients, sarcopenia reflects protein–energy malnutrition, and contains appeal as a method to assess the nutritional status of the patient because of its quantitative, objective and simple methods. Moreover, it has emerged as an independent predictor of poor prognosis in a variety of clinical conditions. Several studies have reported that sarcopenia was associated with worse prognosis, as well as reduced survival, after LT. [10,11] However, it is not always clear whether these consequences were determined from longitudinal studies or simply from cross-sectional studies. In addition, it appears that the consequences can vary according to the definition of sarcopenia. Accordingly, it has been difficult to develop a clear consensus about the prognostic value of sarcopenia in patients with cirrhosis. Moreover, inter-individual differences such as ethnic background may have an impact on sarcopenia. However, there is no consensus on the relationship between sarcopenia and racial classification in patients with cirrhosis.

In the present study, we determined that a systematic review (SR) and meta-analysis (MA) would provide the best and most trustworthy objective analysis of existing evidence. [4,12–18]

To this end, we systemically examined the existing literature regarding all aspects of sarcopenia (low muscle mass) in patients with cirrhosis. We aimed to determine the impact of sarcopenia on prognosis in patients with cirrhosis and the differences between Eastern and Western populations.

## Materials and methods

### Search strategy and data sources

We conducted an independent review of Ovid-MEDLINE (1966 to Jan 2017), EMBASE (1988 to Jan 2017), Web of Science (up to Jan 2017), Cochrane Library (up to Jan 2017), Korean databases such as KoreaMed, the Research Information Service System (RISS), and the Korean Studies Information Service system (KISS). References cited in the text of selected articles were further searched to minimize publication bias. The keywords and Medical Subject Headings (MeSH) were: “sarcopenia”, “muscle mass”, “chronic liver disease”, “CLD”, “cirrhosis”. Boolean operators were also used. The search terms included [((chronic liver disease) OR CLD) OR (liver cirrhosis)] AND [sarcopenia OR (muscle mass)]. This study was conducted according to the Cochrane Handbook for Systematic Reviews of Interventions [19] and the statement by the Preferred Reporting Items for Systematic Reviews and Meta-Analyses group (PRISMA) [20].

### Inclusion and exclusion criteria

**Fig 1:**
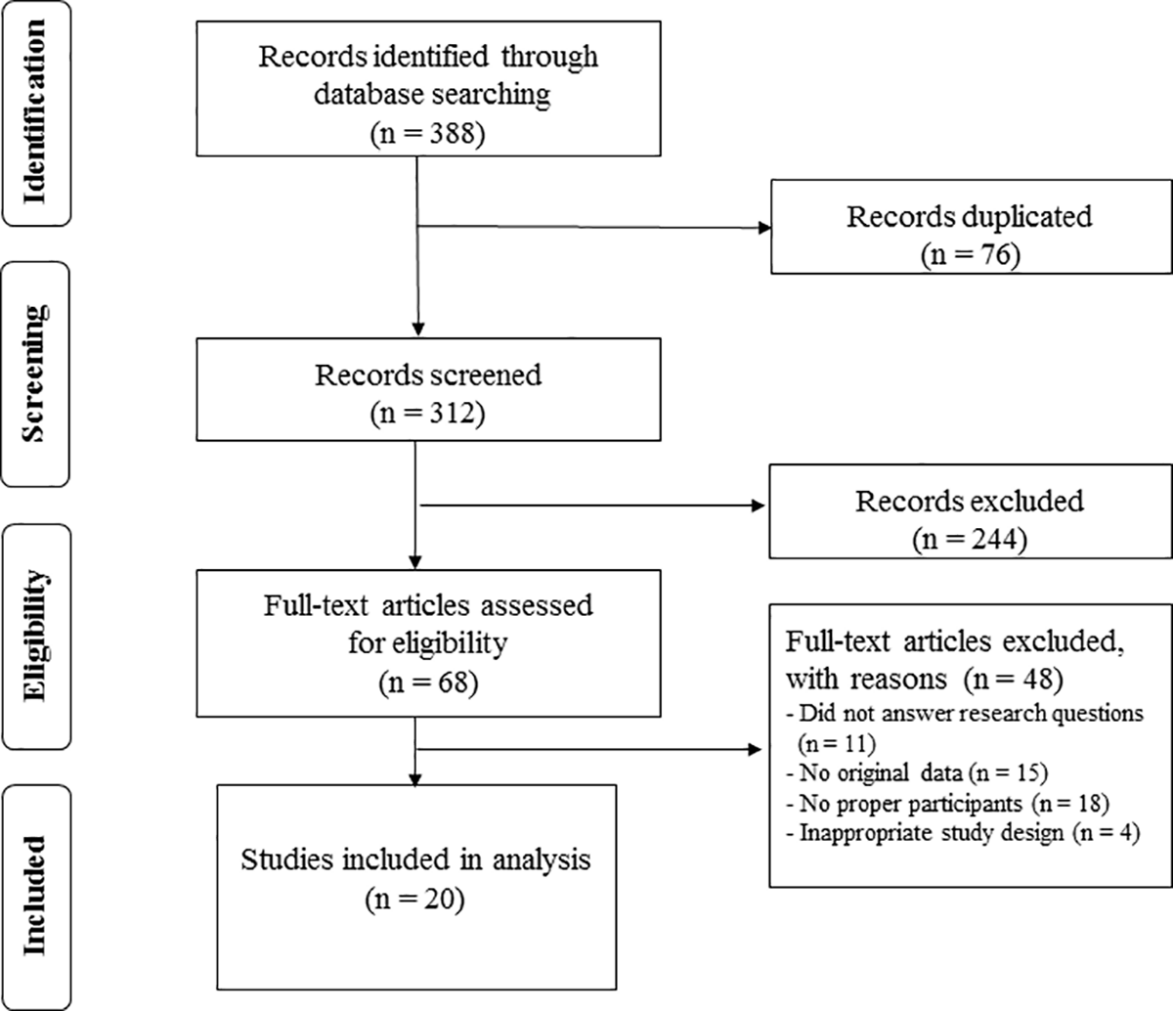
The flow diagram of study selection for the systematic review and meta-analysis.

Studies were included if they met the following criteria: 1) studies were related to sarcopenia and cirrhosis; 2) prospective or retrospective studies; 3) the results included mortality of death; 4) risk estimates included risk ratio, odds ratio or hazard ratio estimates and 95% confidence intervals. All studies included in the review were written in English. We excluded the Animal experiments, chemistry, or cell-line studies and editorial pieces, commentaries, review articles and case reports. Two independent reviewers screened the articles (Kim and Baik). In the first screening the related papers were identified by the titles, abstracts, and text, and the full text of relevant articles was retrieved for validation before final inclusion in the systematic review. A flow diagram of the article selection process is demonstrated in Fig 1.

### Methodological quality assessment

As described in detail previously [15,21], the selected studies were critically appraised using SIGN check list based on the study designs. Studies were rated independently by the first and the second authors. Disagreements were discussed until consensus was reached. The SIGN’s methodological quality assessment tool consists of fourteen internal validity statements and four external validity statements. The tool indicates ‘no’ if the risk of bias is high, ‘yes’ if low, ‘can’t say’ if there is not enough description, and ‘not applicable’ for non-applicable studies. Comprehensively, ‘++’ rating indicates that the study has met major criterion, ‘+’ indicates that most of the criterion are met, and ‘-’ indicates that most criterion are not met.

### Data extraction

The data were extracted in duplicate from all reports and independently recorded on a piloted form by the two of the authors. The following data were extracted for each study: authors; year of publication; country, age, patient selection, details on skeletal muscle mass measurement methods, prevalence of sarcopenia, mortality or survival rate, complications, and length of intensive care unit and hospital stay. Two investigators independently extracted data and differences among reviewers related to data extraction were resolved by discussions and consensus was reached.

### Statistical analyses

All outcomes are reported as in the original articles. A meta-analysis was performed using Comprehensive Meta-Analysis version 2.2 (CMA) and Review Manager 5.3 (The Nordic Cochrane Center, Copenhagen, Denmark). Data are presented as hazard ratio (HR), relative risk (RR) or odds ratio (OR) with 95% confidence intervals (CI). Random effects models were used to calculate summary estimates and to adjust for potential heterogeneity. Studies were weighted according to the inverse of the variance of the HR. Heterogeneity of the included studies was tested using the Higgins I^2^ statistic and meaningful heterogeneity was determined by 50% of the I^2^ value [19,22,23]. Based on the heterogeneity of the included studies, fixed or random effects models were selected to calculate the pooled effect measures. The I^2^ test was included in the forest plots. To assess potential publication bias, Egger’s intercepts for each outcome were also examined.

## Results

### General characteristics of the selected studies

Our initial literature search yielded 388 citations, of which 76 were duplicate studies. Following the screening process, a total of 244 studies were excluded based on the selection criteria, of which 20 studies were ultimately identified as relevant to our review. Therefore, we analyzed 20 studies [24–43] and 4,037 patients (Table 1). A detailed flow chart of the literature search and the study selection is presented in Fig 1.

**Table 1:** Main characteristics and outcomes of included studies.

First author, year, (Ref. no)	Country	No. of Subjects (M:F)	Mean Age (years)	Inclusion	Exclusion	muscle measured	software	level of measure	cutoffs for sarcopenia	Sarcopenia prevalence	RoB
Cruz, 2013 [36]	USA	234 (157:77)	55±9.6	Adults evaluated for LT Patients with liver disease (HBV/HCV, NASH, alcohol, autoimmune/PSC/PBC, fulminant failure)	Patients who received LT for fulminant liver failure	CT scan: CSA (SMI), cm^2^/m^2^	SliceOmatic (Tomovision, Montreal, Quebec, Canada)	L3-L4	M≤52.4 cm^2^/m^2^, F≤38.5 cm^2^/m^2^	70% M 76%	+
DiMartini, 2013 [24]	USA	338 (223:115)	55±10	First time LT without transplantation of other organs Patients with HCC, Patients with liver disease (HCV/HBV, NASH, alcohol, Autoimmune/PSC/PBC, fulminant failure)	N/A	CT scan: CSA (SMI), cm^2^/m^2^	SliceOmatic (Tomovision, Montreal, Quebec, Canada)	L3-L4	M≤52.4 cm^2^/m^2,^ F≤38.5 cm^2^/m^2^	68% M 76% F 51%	+
Durand, 2014 [25]	France	562 (186, 376)	53±8.0	Patients evaluated for LT Patients with cirrhosis (alcohol, HBV/HCV, biliary disease), HCC, refractory ascites	Patients listed for living donor transplantation and multiple organ transplantation, HIV-infected patients	CT scan: Transversal psoas muscle thickness (TPMT)/height, mm/m	N/A	Umbilicus	≤16.8 mm/m	N/A	++
Englesbe, 2010 [26]	USA	163 (103:60)	53.2±9.2	Adult patients undergoing LT Patients with alcoholic cirrhosis, HCC, HCV, PBC, PSC	N/A	CT scan: TPA, mm^2^	MATLAB	L4	Sex-specific tertiles	33% (lowest tertile)	+
Giusto, 2015 [27]	Italy	59 (46:13)	53±12.10 median 59 (range 26–68)	Patients with cirrhosis under evaluation for LT	Patients with acute liver failure, HCC beyond Milan criteria, previous LT, listing for multivisceral or LRLT	CT scan: CSA (SMI), cm^2^/m^2^	Leonardo Syngo	L3-L4	M≤52.4 cm^2^/m^2,^ F≤38.5 cm^2^/m^2^	76% M 78% F 69%	+
Hamaguchi, 2014 [28]	Japan	200 (95:105)	48.78±14.70 median 54 (range 18–69)	Adult patients undergoing LT Patients with HCC, HBV/HCV, PBC/PSC, alcoholic LC, acute liver failure, metabolic liver diseases, Budd-Chiari syndrome	Patients who did not undergo preoperative plain CT imaging	CT scan: TPA, mm^2^	Aquarius NET server	Umblical level	M≤6.7 cm^2^/m^2^ F≤4.1 cm^2^/m^2^	44%	+
Hanai, 2015 [37]	Japan	130 (76:54)	62.75±18.18 median 66 (range 28–91)	Patients with cirrhosis (HBV/HCV, alcohol)	Active malignant disease, HCC, Acute liver failure, renal failure, heart failure, End-stage chronic obstructive lung disease, cirrhotic patients with a serum albumin of ≥3.6 g/dL	CT scan: CSA (SMI), cm^2^/m^2^	SliceOmatic (Tomovision, Montreal, Quebec, Canada)	L3	M≤52.4 cm^2^/m^2^, F≤38.5 cm^2^/m^2^	68% M 82% F 50%	-
Kaido, 2013 [40]	Japan	124	49±14.42 median 54 (range 19–69)	Patients undergoing LT, Patients with HCC, HBV/HCV,PBC/PSC, metabolic liver disease, biliary atresia	Acute liver failure (unable to undergo multifrequency BIA)	Multifrequency BIA: whole body skeletal muscle mass	Inbody 720 (Biospace, Seoul, Korea)	N/A	Less than 90% of the standard level	N/A	+
Kim, 2014 [42]	Korea	65 (41:24)	55±9.2	Patients with cirrhosis (alcohol, viral hepatitis)	Unstable state, Absence of ascites, Creatinine levels above 1.5 times to upper normal limits, Failure of HVPG measurement	CT scan: PMTH(psoas muscle thickness by height, mm/m)	N/A	L4	≤ 14 mm/m	N/A	+
Krell, 2013 [29]	USA	207 (129:78)	51.7	Adult patients undergoing LT, Patients with HCC, HCV/HBV, alcoholic cirrhosis, Autoimmune/PSC/PBC, fulminant hepatitis failure, NASH, Alpha-1 antitryspin deficiency, Wilson's disease	N/A	CT scan: TPA, mm^2^	MATLAB	L4	Sex-specific tertiles	33% (lowest tertile)	+
Lee, 2014 [30]	USA	325 (198:127)	52±9.6	Adult patients undergoing LT, Patients with HCC, HCV, diabetes, hypertension	N/A	CT scan: DMG, TPA, mm^2^	MATLAB	L4, T12	Sex-specific tertiles	33% (lowest tertile)	+
Masuda, 2014 [43]	Japan	204 (103:101)	54 S:53.9±10.5 NS: 54.8±8.5	Patients undergoing LDLT, Patients with HBV/HCV, primary biliary cirrhosis, alcoholic cirrhosis	acute hepatic failure	CT scan: Psoas muscle area, CSA (SMI), cm^2^/m^2^	N/A	L3	M≤800 cm^2,^ F≤380 cm^2^	47% M 58% F 36%	+
Montano-Loza, 2014 [31]	Canada	248 (169:79)	55±1	Patients with cirrhosis (alcoholic, HCV/HBV, alpha-1-antitrypsin deficiency, cryptogenic disease, NASH), HCC	N/A	CT scan: CSA (SMI), cm^2^/m^2^	SliceOmatic (Tomovision, Montreal, Quebec, Canada)	L3	M≤ 53cm^2^/m^2,^ F≤ 41 cm^2^/m^2^	45% M 52% F 30%	++
Montano-Loza, 2012 [32]	Canada	112 (78:34)	54±1	Patients evaluated for LT with cirrhosis(alcoholic, HCV/HBV, autoimmune liver disease), Patients with HCC	N/A	CT scan: CSA (SMI), cm^2^/m^2^	SliceOmatic (Tomovision, Montreal, Quebec, Canada)	L3	M≤52.4 cm^2^/m^2,^ F≤38.5 cm^2^/m^2^	40% M 50% F 18%	+
Tandon, 2012 [33]	Canada	142 (85:57)	52.5±82.87 median 53 (range 47–57)	Adult patients on the LT waiting list Patients with cirrhosis (alcholic, hepatitis C, cryptogenic/NAFLD, autoimmune)	Patients with HCC, acute liver failure, prior LT, Multivisceral LT, LRLT	MRI and CT scans: CSA (SMI), cm^2^/m^2^	SliceOmatic (Tomovision, Montreal, Quebec, Canada)	L3	M≤52.4 cm^2^/m^2,^ F≤38.5 cm^2^/m^2^	41% M 54% F 21%	+
Tsien, 2014 [34]	USA	53 (41:12)	56.9±7.5	Patients undergoing LT Patients with cirrhosis (viral, alcohol, NASH), HCC, colestasis	N/A	CT scan: TPA (PMI), cm^2^/m^2^	Leonardo Workstation using oncocare	L4	PMI<50years M≤12.3 cm^2^/m^2,^ F≤10.5 cm^2^/m^2^ PMI>50years M≤10.1 cm^2^/m^2^, F≤10.3 cm^2^/m^2^	62%	++
Waits, 2014 [41]	USA	348 (215:133)	51±10.0	Adults patients who received LT Patients with HCC, HCV, portal hypertension/cirrhosis, diabetes mellitus, hypertension	N/A	CT scan: morphometric age(including TPA, PD)	MATLAB 13.0	L4	Morphometric age	N/A	++
Yadav, 2015 [35]	USA	213 (129:84) (S 47, NS 165)	55.3±8.6	All patients listed for LT Patients with HCC, liver disease (alcoholic, HCV, PBC/PSC, NASH, cryptogenic)	Patients with hepatopumonary syndrome, protopulmonary hypertension, LT candidates without abdominal CT imaging	CT scan: CSA (SMI), cm2/m2	SliceOmatic (Tomovision, Montreal, Quebec, Canada)	L3	M≤52.4 cm^2^/m^2,^ F≤38.5 cm^2^/m^2^	22% M 28.1% F 13.1%	+
Hanai, 2016 [38]	Japan	149 (82:67)	61.5±17.31 median 65 (range, 28–88)	Patients with cirrhosis	Patients with HCC, acute liver failure, heart failure, or end-stage chronic obstructive lung disease at entry	CT scan: CSA (SMI), cm2/m2	SliceOmatic (Tomovision,Montreal, Quebec, Canada)	L3	M≤52.4 cm^2^/m^2^, F≤38.5 cm^2^/m^2^	63.1%	+
Hara, 2016 [39]	Japan	161 (94:67)	67±9	Patients with cirrhosis (HCV, HBV, alcoholic), patients with HCC	N/A	Multifrequency BIA: whole body skeletal muscle mass	InBody 720 (Biospace, Seoul, Korea)	N/A	M≤1.7 kg/m^2^, F≤1.2 kg/m^2^	24.80%	+

NASH; Non-alcoholic steatohepatitis, PSC; primary sclerosing cholangitis, LT; liver transplantation, MAMC, mid-arm muscle circumference; DEXA, dual-energy X-ray absorptiometry; FFMI, fat-free mass index; APMT, axial psoas muscle thickness; AWMA, abdominal wall muscle area; AWMI, abdominal wall muscle index; CSA, cross-sectional area; HU, Hounsfield units; HCC, hepatocellular carcinoma; IMAC, intramuscular adipose content (defined as region of interest of multifidus muscle [Hounsfield units] divided by region of interest of subcutaneous fat [Hounsfield units]); L3, third lumbar vertebra; L4, fourth lumbar vertebra; LDLT, living donor liver transplantation; LRLT, living related liver transplantation; LT, liver transplantation; N/A, not available;NS, patients without sarcopenia; OLT, orthotropic liver transplantation; PD, psoas density; PMI, psoas muscle index (cm2/m2); PSMA, paraspinal muscle area; PSMI, paraspinal muscle index; S, patients with sarcopenia; SMI, skeletal muscle index (cm2/m2); SMK, skeletal muscle index; T12, 12th thoracic vertebra; TPA, total psoas area; TPMT, transversal psoas muscle thickness; TPV, total psoas volume; ICC, IMAC, intramuscular adipose tissue contentp PMI, psoas muscle mass index

All twenty studies analyzed in this article has been implemented since 2010 and published between 2010 and 2016, and the mean age of the participants were 54.78 years (range, 48.8–67.0). Seven studies [28,37–40,42,43] included Asian participants including Korean and Japanese, and the remaining 13 studies [25–27,29–36,41] included Westerns including American, Italian, French, and Canadian. Most of the participants were liver disease patients (alcohol, NASH, HBV/HCV, HCC, cirrhosis, and so on) and were waiting for LT or have received LT. Participants was excluded if then had other organ transplantation besides liver or had liver failure (Table 1).

### Methodological quality and risk of bias in the included studies

According to the quality evaluation of individual studies included in this article (Fig 1), all of the studies clarified their research objectives. 43% of the researches did not address the attrition bias, but all studies displayed low risk of bias in terms of selection, performance, and detection. In addition, there were 4 studies [25,31,34,41] with ‘++’ rank in general methodological quality of study, 15 studies [26–30,32,33,35,36,38–40,42,43] with a ‘+’ rank, and one study [37] with a ‘–‘ rank, indicating that more than 95% of the studies had achieved ‘+’ rank or more (Table 1). Therefore, most of the studies included in the review were classified as studies with low risk of bias, indicating high quality in general.

### Outcomes

#### Prevalence of sarcopenia in cirrhosis

The definitions of sarcopenia among research are as follows: 2 studies [39,40] used bioelectrical impedance analysis (BIA), 10 studies [31–36,38–40,43] used cross–sectional muscle area with corresponding skeletal muscle index (SMI, value normalized by the square of the height), and 8 studies [25,26,28–30,34,41,42] used total psoas muscle area (TPA). For the studies that used SMI as criteria, the cut-off of sarcopenia was ≤ 52.4–53.0 cm^2^/m^2^ for men and ≤ 38.5–41.0 cm^2^/m^2^ for women. For BIA, the cut-off was below 2 standard deviations. Most of the studies used CT to diagnose sarcopenia (Table 1).

The prevalence rate of sarcopenia among participants was mean 48.1% (range, 24.8–70.0%), and appeared more among men with a rate of 61.6% (range, 28.1–82.0%) whereas the rate was 36% (range, 13.1–69.0%) for women (Table 1).

#### Clinical impact of sarcopenia on mortality or survival in cirrhosis

**Fig 2:**
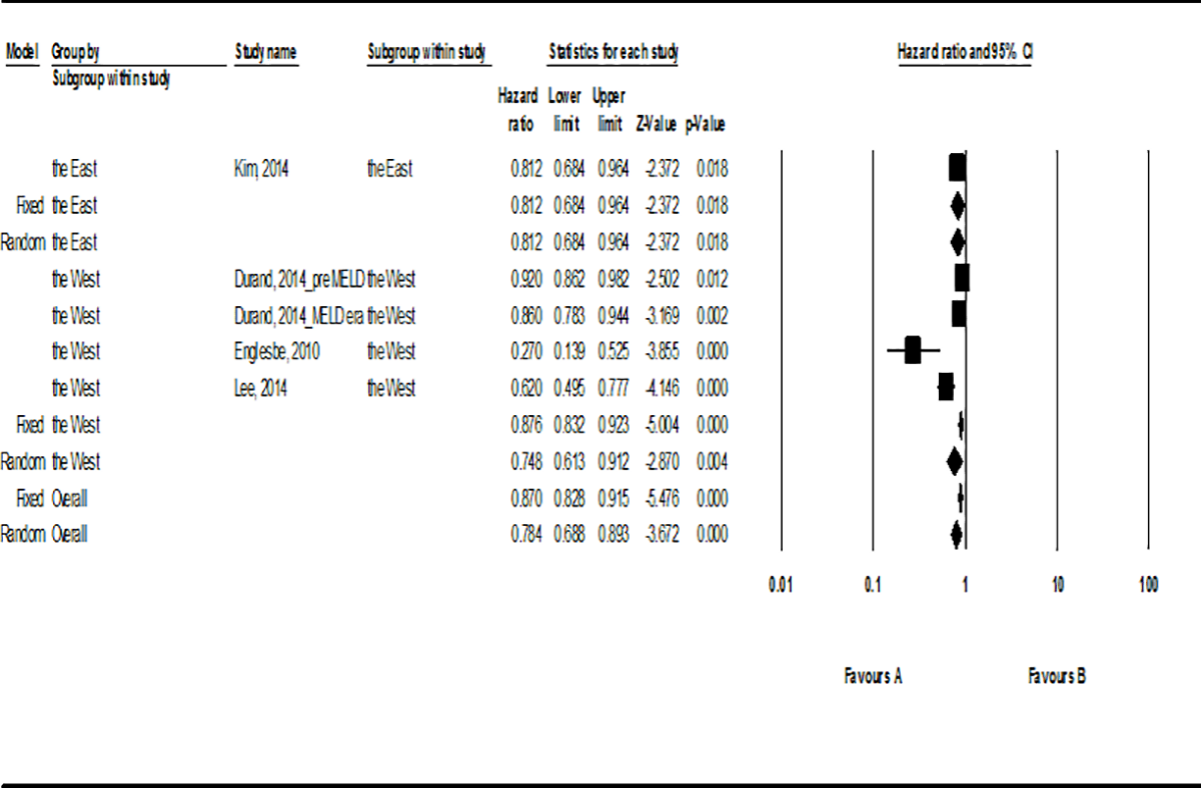
Forest plot for the mortality in accordance to muscle mass.

The hazard ratio (HR) of mortality in accordance to the participants’ muscle mass was 0.78 (95% CI, 0.68–0.89; *P* < .001), implying that mortality decreases at a statistically significant rate of 22% for higher muscle mass (Fig 2). There was possibility of publication bias (Egger’s regression intercept, -3.90; *P* < .01) (Table 2) and the heterogeneity of the literatures were high (I^2^ = 83.32%). According to the subgroup analysis based on the race of the participants, Asians had HR 0.81 (95% CI, 0.68–0.96; *P* < .05) whereas Westerns had HR 0.75(95% CI, 0.61–0.91; *P* < .05) (Fig 2).

**Table 2:** Egger test results of studies.

Egger’s regression intercept	
Mortality in accordance to the participants’ muscle mass	
*Intercept*	-3.90296
*Standard error*	0.57924
*95% lower limit (2-tailed)*	-5.74636
*95% upper limit (2-tailed)*	-2.05956
*t-value*	6.73808
*df*	3.00000
*p-value (1-tailed)*	0.00334
*p-value (2-tailed)*	0.00668
The odds ratio of mortality for the sarcopenia group	
*Intercept*	1.88203
*Standard error*	7.26106
*95% lower limit (2-tailed)*	-29.35980
*95% upper limit (2-tailed)*	33.12386
*t-value*	0.25919
*df*	2.00000
*p-value (1-tailed)*	0.40986
*p-value (2-tailed)*	0.81972
The hazard ratio of mortality for the sarcopenia group	
*Intercept*	1.87088
*Standard error*	0.37908
*95% lower limit (2-tailed)*	0.89643
*95% upper limit (2-tailed)*	2.84532
*t-value*	4.93535
*df*	5.00000
*p-value (1-tailed)*	0.00217
*p-value (2-tailed)*	0.00434

**Fig 3:**
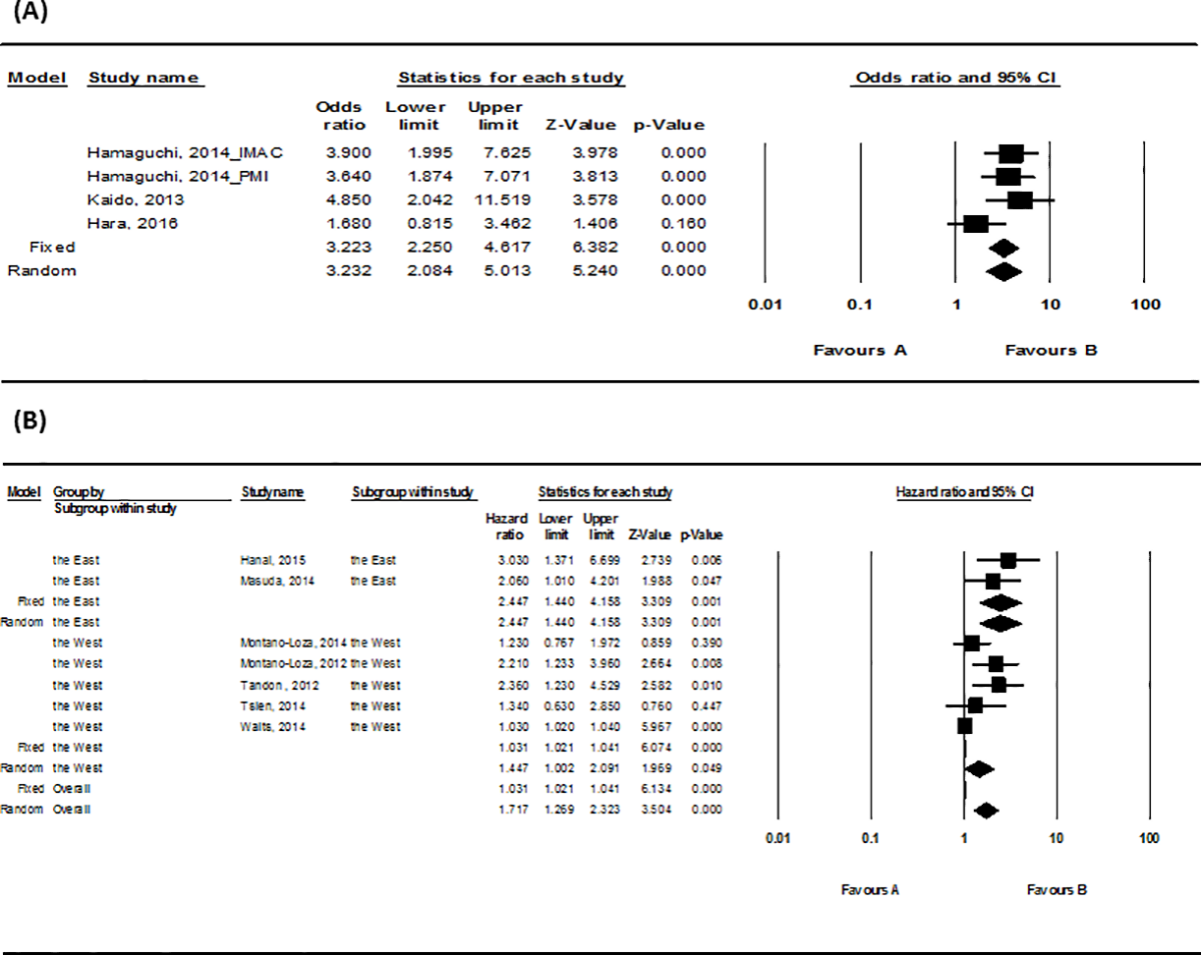
Forest plot for the mortality for the sarcopenia group. (A) The odds ratio (OR) of mortality for the sarcopenia group (B) The hazard ratio (HR) of mortality for the sarcopenia group.

The odds ratio (OR) of mortality was 3.23(95% CI, 2.08–5.01; *P* < .001) for the sarcopenia group, which implies a 3.23 times higher mortality rate compared to the non-sarcopenia group (Fig 3A). The result was statistically significant. The heterogeneity of the literature was low (I^2^ = 32.05%) and there was not a possibility of a publication bias (Egger’s regression intercept, 1.882; *P* = .40) (Table 2).

The hazard ratio (HR) of mortality for the sarcopenia group was 1.72(95% CI, 1.27–2.32; *P* < .001) (Fig 3B). This indicates that the sarcopenia group had 1.72 times higher mortality compared to the non-sarcopenia group, and the result was statistically significant. There was possibility of publication bias (Egger’s regression intercept, 1.870; *P* < .005) (Table 2) and the heterogeneity of the study was quite high (I^2^ = 75.5%). According to the analysis of each race subgroup, Asians had a HR 2.45(95% CI, 1.44–4.16; *P* = .001; I^2^ = 0) whereas Westerners had a HR 1.45(95% CI, 1.002–2.09; *P* < .05; I^2^ = 70%) (Fig 3B). Asian participants had higher mortality related to sarcopenia compared to the Western participants.

#### Impact on the post-transplant infection

**Fig 4:**
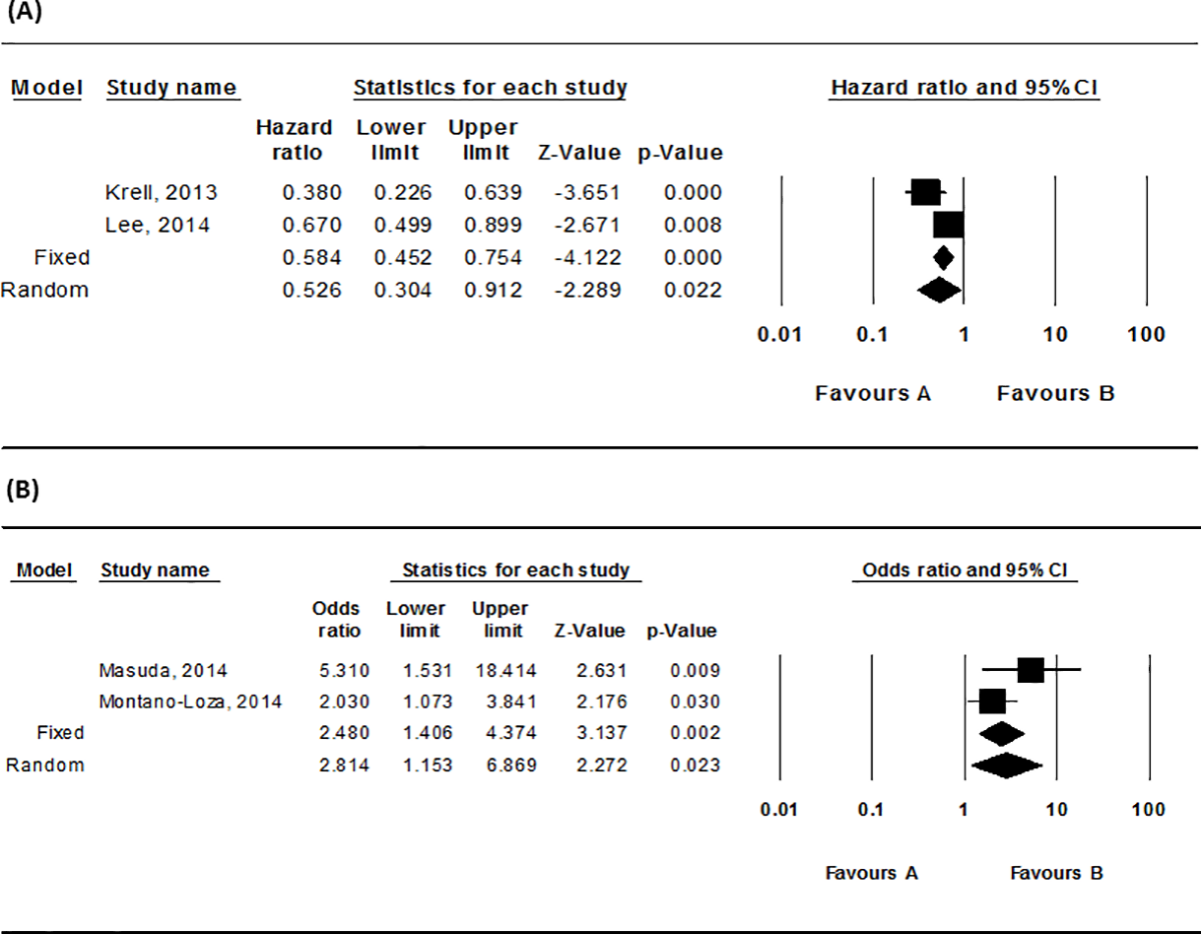
Forest plot for the complications occurrence. (A) The HR of complications occurrence such as severe infection (B) The HR of complications occurrence such as sepsis or severe infection to sarcopenia.

HR of complications occurrence such as severe infection to muscle mass was 0.53(95% CI, 0.30–0.91; *P* < .05; I^2^ = 71.17%) (Fig 4A). Higher muscle mass implied a statistically significant reduction of 47% in complication occurrence, but the heterogeneity of the article was quite high (Table 2).

Also, HR of complications occurrence such as sepsis or severe infection to sarcopenia was 2.81(95% CI, 1.15–6.87; *P* < .05), implying a 2.8 times higher complication occurrence for the sarcopenia group compared to the non-sarcopenia group, and the result was statistically significant (Fig 4B). The heterogeneity of the article was low (I^2^ = 45.0%) (Table 2).

#### Impact on the length of hospitalization

Two [24,31] the length of stays (ICU, hospital days) related to sarcopenia, and the length was longer for the sarcopenia group than the non-sarcopenia group. The finding was statistically significant.

### Publication bias

Publication bias was assessed using Egger's (Table 2) and Begg's tests (S1 Fig). On the basis of these statistical tests, there was no evidence of publication bias in the odds ratio of mortality for the sarcopenia group (intercept = 1.88, p = .40). However, statistical tests suggested that there might be publication bias mortality in accordance to the participants’ muscle mass (intercept = -3.90, p < .01) and the hazard ratio of mortality for the sarcopenia group (intercept = 1.87, p < .005), respectively.

## Discussion

This meta-analysis of 20 studies including 4,037 patients aimed to determine the predictive value of sarcopenia for prognosis in patients with cirrhosis. According to the results, there is consistent evidence that sarcopenia is associated with lower survival in patients with cirrhosis, independent of other risk factors such as age and MELD score. In addition, sarcopenia was frequently associated with increased risk of infection and increased length of hospitalization. We also assessed whether ethnicity influence the association because most studies were carried out in various geographical locations. To our knowledge, this is the first meta-analysis investigating the association between ethnic differences and the impact of sarcopenia on the mortality in liver cirrhosis. We found that sarcopenia in the Asian populations was associated with higher mortality compared to Western populations.

In patients being evaluated for or awaiting liver transplantation, sarcopenia was associated with poorer survival in seven studies [25,32,33,37–39,42], whereas two other studies showed no significant association [27,35]. In patients who underwent liver transplantation, eight studies showed an association between sarcopenia and post-transplantation survival [24,26,36], whereas three found no association. As described in detail previously [44], this systematic review also revealed that the diversity and complexity of the measurement methods used for the diagnosis of sarcopenia. For example, two studies that used bioelectrical impedance analysis (BIA) in patients undergoing or awaiting liver transplantation showed that low skeletal muscle mass was an independent risk factor for mortality. [39,40] Although eighteen other studies estimated the skeletal muscle mass from abdominal cross-sectional images using CT, which is a great methods for diagnosing sarcopenia, measurement techniques for muscle area varied including the L3-4 skeletal muscle index, the psoas muscle area and the dorsal muscle area. Moreover, the definition of sarcopenia in patients with cirrhosis lacks a consensus regarding adequate cut-off values. Most studies defined sarcopenia using the L3 SMI cut-off values suggested by Prado (L3 SMI: ≤ 38.5 cm^2^/m^2^ for women and ≤ 52.4 cm^2^/m^2^ for men). [45] Therefore, the cut-off values may not be optimal for prognostication of patients with cirrhosis, who differ from cancer patients. Moreover, this could have led to insufficient classification of Asian patients, because the cut-off values suggested by Prado were determined using stratification analysis for low muscle mass and mortality in 250 obese Canadian patients. [45] According to our analysis of reports separated into Western population-based studies and Asian population-based studies, sarcopenia in Asian populations had more impact on mortality than sarcopenia in Western populations. These results can be attributed to differences in racial characteristics, body size, dietary regimes, and life quality between Asian and Western individuals in different countries. Previous studies reported that the mean muscle mass of Asians is apporoximately 15% lower than that of Westerners even after height adjustments. [46,47] Few current studies have proposed methods for the evaluation and measurement of sarcopenia in people of other ethnicities. Consequently, establishing criteria for evaluating and measuring sarcopenia in diverse ethnicities is essential.

This systematic review suggests that sarcopenia is an important prognostic factor, independent of MELD and CTP scores. Currently, patients with the highest MELD scores are prioritized during the allocation of donor livers, because the MELD score remains strongly associated with waiting list mortality. However, 71% of patients who died on the waiting list had a MELD score ≤25 at registration. [48,49] The study by Durand et al showed that the MELD-sarcopenia score, which combines MELD and psoas muscle area scores, is superior to that of the MELD score. These findings suggest that sarcopenia is an attractive prognostic factor to improve organ allocation in patients with cirrhosis. However, Tandon et al noted that the impact of sarcopenia was significant in patients with low MELD scores (<15; *P* = .02) but not in patients with higher MELD scores (≥15; *P* = .59). [33] These results are consistent with data from Merli et al, who demonstrated that muscle loss was predictive of mortality in CTP class A and CTP class B patients but not in patients with CTP class C cirrhosis. [50] Taken together, these results suggest that further validation is needed. If validated, clinical trials are warranted to explore whether transplantation in sarcopenic patients with lower MELD scores may be superior.

According to our analysis, despite inconsistencies, several lines of evidence suggest that sarcopenia is associated with longer hospital stay and higher rates of infections after transplantation. Interestingly, the study by Montano et al showed that the higher mortality risk in cirrhotic patients with sarcopenia seems to be related to a higher frequency of sepsis-related death and not to liver failure mortality. [32]

Sarcopenia seems to be the result of complex interactions involving inadequate nutrition, impaired synthesis of glycogen, underlying hypermetabolism, and impairment of skeletal muscle protein synthesis due to portosystemic shunting in cirrhotic liver. [51–54] Although the mechanisms by which sarcopenia leads to poor outcomes in patients with cirrhosis have not been completely clarified, several hypotheses have been suggested. First, oxidative pathways are altered in skeletal muscle during muscle wasting and this is likely a consequence of mitochondrial abnormalities. [55] In addition, the skeletal muscle is a secretory organ of cytokines and other peptides that have autocrine, paracrine, or endocrine actions and are extensively involved in inflammatory processes. [56] An understanding of the underlying mechanisms of sarcopenia with cirrhosis is necessary to better develop treatment strategies.

There were some limitations of the present study that require further discussion. First, the characteristics of the included studies were not completely consistent, including patient characteristics, etiologies of cirrhosis, and methodology. Second, the limited number of studies that were included and the relatively small pooled sample size also imposed a limitation on our analysis. Third, all included studies were retrospective, observational cohort studies. Although this may have resulted in selection bias, the study cohorts consisted of nonselected, consecutive patients. Fourth, recent consensus recommends using the presence of both low muscle mass and low muscle function for the definition of sarcopenia. However, we analyze the prognostic value of only low skeletal muscle mass because muscle mass alone has been extensively used to define sarcopenia in most studies heretofore. Sarcopenia is already a widely recognised term, so replacing it might lead to further confusion. Therefore, accurate and universal definition of sarcopenia is needed. Finally, we only included studies written in English and therefore may have missed studies that were published in other languages and not indexed in the databases that were searched in this study.

In summary, regardless of which of the three assessment methods (i.e., BIA, SMI or TPA on CT scans) is used to define sarcopenia, sarcopenia is associated with poor prognosis including higher risk of mortality in patients with cirrhosis. Furthermore, Asian populations had higher mortality related to sarcopenia compared to Western populations. However, the definition of sarcopenia remains controversial and multiple definitions have been used in the literature. Therefore, further prospective studies are needed to clarify diagnostic methods to standardize muscularity assessment and definitions according to the ethnicity, gender, and age to reflect individual health status.

## Supporting information

S1 Table PRISMA 2009 check list. (DOC)

S1 Fig Funnel plot of standard error by log hazard ratio or odds ratio. Each point represents a separate study for the indicated association.
(DOCX)

S2 Fig PRISMA 2009 flow diagram. (DOC)
