# Supplementary material for: Prognostic value of sarcopenia in patients with liver cirrhosis: A systematic review and meta-analysis
Source: PLoS One. 2017 Oct 24;12(10):e0186990. doi: 10.1371/journal.pone.0186990 (PMC5655454; doi:10.1371/journal.pone.0186990)
Supplement: S1 Fig — Each point represents a separate study for the indicated association. (DOCX) [file pone.0186990.s002.docx]

|  |  |
| --- | --- |
| A) The hazard ratio of mortality in accordance to the participants’ muscle (plot observed studies only) | A) The hazard ratio of mortality in accordance to the participants’ muscle (plot observed studies and imputed) |
|  |  |
| B) The odds ratio of mortality (plot observed studies only) | B) The odds ratio of mortality (plot observed studies and imputed) |
|  |  |
| C) The hazard ratio of mortality in sarcopenia group (plot observed studies only) | C) The hazard ratio of mortality in sarcopenia group (plot observed studies and imputed) |

**Supplementary Fig 1.** Funnel Plot of Standard Error by Log Hazard Ratio or Odds Ratio. Each point represents a separate study for the indicated association.
