# Supplementary material for: Prognostic value of sarcopenia in patients with liver cirrhosis: A systematic review and meta-analysis
Source: PLoS One. 2017 Oct 24;12(10):e0186990. doi: 10.1371/journal.pone.0186990 (PMC5655454; doi:10.1371/journal.pone.0186990)
Supplement: S2 Fig — (DOC) [file pone.0186990.s003.doc]

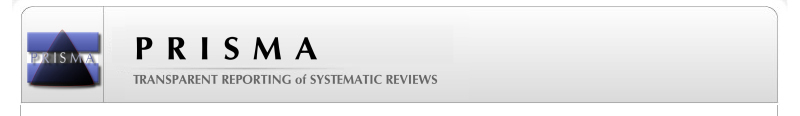
**PRISMA 2009 Flow Diagram**

**Screening**

**Included**

**Eligibility**

**Identification**

Records identified through database searching
(n =388)

Additional records identified through other sources
(n = 0)

Records after duplicates removed
(n =312)

Records screened
(n =312)

Records excluded
(n =244)

Full-text articles assessed for eligibility
(n =68)

Full-text articles excluded, with reasons
(n =48)

Studies included in qualitative synthesis
(n = 20)

Studies included in quantitative synthesis (meta-analysis)
(n = 20)
